# An Experimental Pre-Post Study on the Efficacy of Respiratory Physiotherapy in Severe Critically III COVID-19 Patients

**DOI:** 10.3390/jcm10102139

**Published:** 2021-05-15

**Authors:** Denise Battaglini, Salvatore Caiffa, Giovanni Gasti, Elena Ciaravolo, Chiara Robba, Jacob Herrmann, Sarah E. Gerard, Matteo Bassetti, Paolo Pelosi, Lorenzo Ball, on behalf of the GECOVID Group

**Affiliations:** 1 Anesthesia and Intensive Care, San Martino Policlinico Hospital, IRCCS for Oncology and Neuroscience, 16132 Genoa, Italy; giovanni.gasti@gmail.com (G.G.); eleciaravolo91@gmail.com (E.C.); kiarobba@gmail.com (C.R.); ppelosi@hotmail.com (P.P.); lorenzo.ball@unige.it (L.B.); 2 Department of Medicine, University of Barcelona (UB), 08007 Barcelona, Spain; 3 Intensive Care Respiratory Physiotherapy, Rehabilitation and Functional Education, San Martino Policlinico Hospital, IRCCS for Oncology and Neurosciences, 16132 Genoa, Italy; salvatore.caiffa@hsanmartino.it; 4 Department of Surgical Sciences and Integrated Diagnostics (DISC), University of Genoa, 16132 Genoa, Italy; 5 Department of Biomedical Engineering, Boston University, Boston, MA 02215, USA; jakeherr@bu.edu; 6 Department of Radiology, University of Iowa, Iowa City, IA 52242, USA; sarah-gerard@uiowa.edu; 7 Infectious Diseases Unit, San Martino Policlinico Hospital, IRCCS for Oncology and Neurosciences, 16132 Genoa, Italy; matteo.bassetti@hsanmartino.it

**Keywords:** COVID-19, chest physiotherapy, lung ultrasound, intensive care unit, SARS-CoV-2, respiratory physiotherapy, rehabilitation

## Abstract

*Background*: Respiratory physiotherapy (RPT) is considered essential in patients’ management during intensive care unit (ICU) stay. The role of RPT in critically ill COVID-19 patients is poorly described. We aimed to investigate the effects of RPT on oxygenation and lung aeration in critically ill COVID-19 patients admitted to the ICU. *Methods*: Observational pre-post study. Patients with severe COVID-19 admitted to the ICU, who received a protocolized CPT session and for which a pre-and post-RPT lung ultrasound (LUS) was performed, were included. A subgroup of patients had an available quantitative computed tomography (CT) scan performed within 4 days from RPT. The primary aim was to evaluate whether RPT improved oxygenation; secondary aims included correlations between LUS, CT and response to RPT. *Results*: Twenty patients were included. The median (1st–3rd quartile) PaO_2_/FiO_2_ was 181 (105–456), 244 (137–497) and 246 (137–482) at baseline (T0), after RPT (T1), and after 6 h (T2), respectively. PaO_2_/FiO_2_ improved throughout the study (*p* = 0.042); particularly, PaO_2_/FiO_2_ improved at T1 in respect to T0 (*p* = 0.011), remaining higher at T2 (*p* = 0.007) compared to T0. Correlations between LUS, volume of gas (rho = 0.58, 95%CI 0.05–0.85, *p* = 0.033) and hyper-aerated mass at CT scan (rho = 0.54, 95% CI 0.00–0.84, *p* = 0.045) were detected. No significant changes in LUS score were observed before and after RPT. *Conclusions*: RPT improved oxygenation and the improvement persisted after 6 h. Oxygenation improvement was not reflected by aeration changes assessed with LUS. Further studies are warranted to assess the efficacy of RPT in COVID-19 ICU patients.

## 1. Introduction

In late December 2019, an outbreak caused by severe acute respiratory syndrome coronavirus 2 (SARS-CoV-2) started from the city of Wuhan, China and rapidly spread worldwide [1]. Patients with SARS-CoV-2 present with a broad spectrum of symptoms, including severe hypoxemic respiratory failure requiring mechanical ventilation and possible multiorgan failure in most severe cases [2,3]. Distinct respiratory phenotypes characterize coronavirus disease (COVID-19), making ventilatory management particularly challenging [4,5].

Respiratory physiotherapy (RPT) is an essential part of patient management and rehabilitation during intensive care unit (ICU) stay [6], as it improves physical function and clinical outcomes in general ICU patients [7]. There is no consensus on the efficacy of RPT in the specific setting of COVID-19 and the burden of ICU resources utilization associated with the emergency context of the pandemic might have resulted in an undervaluation of its role. In fact, the role of RPT in COVID-19 is poorly described and studies on its efficacy are warranted [6,8,9,10,11,12,13,14].

Lung ultrasound (LUS) can estimate lung aeration and it has been used in critically ill patients as a tool for respiratory monitoring at the bedside and to better identify patients who can benefit from specific treatments such as alveolar recruitment maneuvers (RMs), positive end-expiratory pressure (PEEP), RPT and prone positioning [8]. In COVID-19 patients, LUS has been extensively used [15,16,17] and proposed as a tool to stratify patients’ risk and to monitor the course of the disease [18].

The primary aim of this study was to evaluate the effectiveness of RPT in improving oxygenation in severe critically ill COVID-19 patients admitted to ICU. Secondary aims included associations between response to RPT and computed tomography (CT) quantitative analysis and changes in LUS score. We hypothesized that RPT improves gas exchange and that CT and LUS are associated with different degrees of oxygenation improvement.

## 2. Materials and Methods

### 2.1. Study Design and Population

This was an observational study with a pre–post design conducted in a 39-bed ICU in a university-affiliated hospital in Genoa, Italy, from 29th February 2020 to 30th June 2020. Approval for this study was obtained from the local Ethic Committee (Comitato Etico Regione Liguria, registry number 163/2020) who waived informed consent for retrospectively collected data. According to local regulations, consent was delayed after recovery of consciousness for prospectively collected data in unconscious patients.

### 2.2. Inclusion and Exclusion Criteria

**Figure 1:**
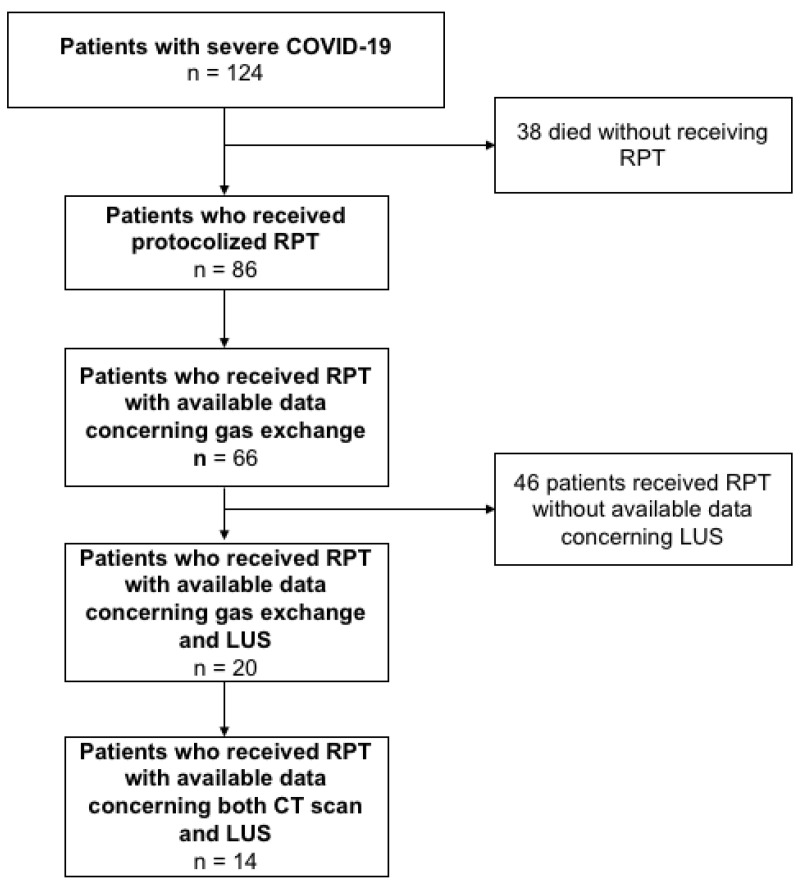
From 29th February 2020 to 30th June 2020, 124 patients confirmed positive for SARS-Cov-2 infection were admitted to the ICU. At ICU admission, all patients presented intubated and mechanically ventilated. Thirty-eight patients died before receiving respiratory physiotherapy (RPT), while eighty-six patients started RPT when deemed ready. Data concerning gas exchange during RPT were available for 66 patients, while data concerning lung ultrasound (LUS) imaging during RPT were available for 20 patients, of whom 14 presented a concomitant computed tomography (CT) scan performed within a few days from the RPT session.

All consecutive adult patients admitted to the ICU due to severe COVID-19-related acute hypoxemic respiratory failure were screened for enrollment. The following inclusion criteria were considered: severe COVID-19 patients [19] admitted to the ICU intubated and mechanically ventilated, with age ≥18 years old and confirmed positive for SARS-CoV-2 infection by reverse transcription-polymerase chain reaction (RT-PCR) on nasopharyngeal swab specimens at ICU admission who underwent respiratory physiotherapy during ICU course, and with available data concerning RPT, gas exchange and LUS before and after RPT. To assess the generalizability of our findings, we also collected gas exchange data of patients that received RPT without available CT and LUS data. Figure 1 represents the patients’ inclusion flow.

### 2.3. Data Collection and Definitions

Patient demographic characteristics were collected at study entry, including: age, gender, body mass index (BMI in kg/m^2^), previous chronic comorbidities, hypertension, diabetes mellitus, cancer, chronic respiratory disease (defined as asthma or chronic obstructive pulmonary disease), end-stage renal disease (defined as estimated glomerular filtration rate <15 mL/min/1.73 m^2^), moderate/severe liver disease (defined as compensated/decompensated liver cirrhosis), chronic neurological disease (defined as previous neurological disease), chronic cardiovascular disease, active smoker, and sequential organ failure assessment (SOFA) score [20].

**Figure 2:**
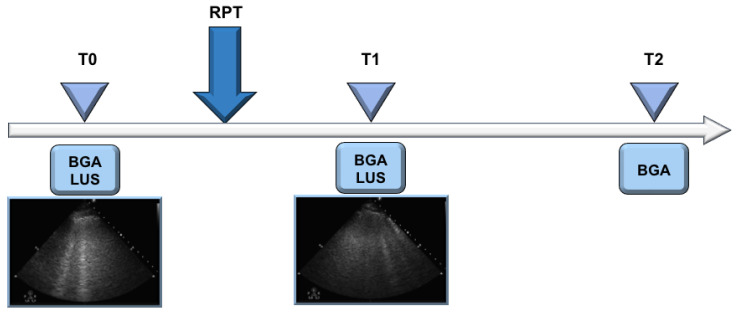
This figure represents the three timepoints of our pre-post study. At baseline/before RPT (T0) a lung echography (LUS) and a blood gas analysis (BGA) were assessed, followed by a RPT protocolized session; then, a new LUS and BGA immediately after RPT (T1) and 6 h thereafter (T2) were collected.

Arterial blood gas analysis, including arterial partial pressure of oxygen (PaO_2_), arterial partial pressure of carbon dioxide (PaCO_2_), pHa, serum bicarbonate, PaO_2_ to fraction of inspired oxygen (FiO_2_) ratio, lung ultrasound (LUS), ventilatory management including type of ventilatory support, PEEP in cmH_2_O, pressure support, respiratory rate in breaths per minutes, tidal volume, mean arterial pressure (MAP) and hearth rate were collected at three time points: before physiotherapy (T0), after physiotherapy (T1) and 6 h thereafter (T2). All these data were collected the first day RPT started. Figure 2 shows the study time-points. Before starting RPT (T0), we assessed a blood gas analysis and LUS; then, the patients underwent a RPT session immediately followed by a blood gas analysis and LUS (T1); and finally, 6 h thereafter, each patient received a blood gas analysis (T2).

### 2.4. Respiratory Physiotherapy

At ICU admission, all patients were intubated, deeply sedated and curarized to allow invasive mechanical ventilation. Once the patient was judged ready to initiate the weaning phase, RPT was always performed.

In COVID-19 patients free from sedation and curarization but still mechanically ventilated through a tracheostomy cannula in assisted mode (pressure support mode), RPT was started, and continued daily with the same protocolized maneuvers until weaning from the ventilator.

After the weaning phase, all COVID-19 patients who were breathing spontaneously received a conventional oxygen therapy (COT) delivered with Venturi masks, oxygen nasal cannulas or tracheostomy and received a daily session of protocolized RPT for spontaneously breathing patients until ICU discharge.

Respiratory physiotherapy maneuvers were performed by one experienced physiotherapist specialized in RPT for critically ill patients (SC), with two protocols in patients receiving assisted invasive mechanical ventilation or spontaneously breathing patients after weaning and extubation receiving COT.

#### 2.4.1. RPT Protocolized Maneuvers in COVID-19 Patients in Pressure Support Mode

Once the patient was judged ready to start the weaning phase, RPT was initiated during assisted mechanical ventilation (pressure support mode) through a tracheostomy.

During respiratory physiotherapy, all patients underwent bed head elevation (30°) and early passive mobilization, with the following respiratory maneuvers:

(1) Subglottic secretion drainage;
(2) Manual assisted cough (at zero PEEP);
(3) Assisted alveolar recruitment consisting in active cycle of breathing technique;
(4) Right lateral to left lateral positioning.

This RPT protocolized session was performed once daily, with a median duration of 30 min per day continuously, until the day of extubation. The RPT session was performed for 4 days (1–4) before weaning from the ventilator.

#### 2.4.2. RPT Protocolized Maneuvers in COVID-19 Patients Breathing Spontaneously

After weaning from the ventilator, each spontaneously breathing patient receiving COT underwent RPT with bed head elevation (30°), early sitting position, active mobilization, and the following respiratory maneuvers:

(1) Airways cleaning techniques;
(2) Manual stimulated cough and sputum induction;
(3) Manual alveolar recruitment consisting in EzPAP operated a zero PEEP (Portex, Smiths Medical, London, UK) and active cycle of breathing technique.

All the above mentioned protocolized techniques have been previously described more specifically by our group [8].

This RPT session was performed once daily, from the day of weaning from the ventilator to the day of ICU discharge, for a median duration of each session of 30 min.

### 2.5. Lung Imaging

Lung ultrasound was performed immediately before and after RPT by two experienced operators (D.B., S.C.); controversies were solved by a third operator. LUS was performed using a convex or linear transducer connected to a Philips Sparq^®^ ultrasound machine according to the patient’s body size, using a single-focal point modality and setting the focal point on the pleural line. Twelve areas were investigated: anterior midclavicular (superior and inferior) right and left, posterior paraspinal (superior and inferior) right and left, lateral axillary (superior and inferior) right and left (Figure S1). Details on the LUS score are described in the Supplemental Material (SM) [21].

When available, we collected CT scans performed for clinical indication within 4 days from the RPT session. Scans were acquired in expiratory breath-hold on a Somatom Definition Flash (Siemens^®^, Erlangen, Germany). Images were segmented using an automated neural network [22,23] followed by manual refinement. Quantitative lung CT analysis was based on commonly used aeration thresholds to discriminate hyper-, normally, poorly and non-aerated lung tissue [24]. Lung volume, weight, and gas volume were also computed.

### 2.6. Statistical Analysis

A formal sample size calculation was difficult due to the lack of previous data in this setting. However, assuming a PaO_2_/FiO_2_ ratio at baseline of 150 ± 50 mmHg and a strong intra-patient correlation (R = 0.8), we needed to include at least 15 patients to achieve 80% power to detect a 10% increase in PaO_2_/FiO_2_ after RPT at an alpha level of 5%.

Student’s t-test or Mann–Whitney U-tests were used to compare continuous variables and chi-square tests were used for categorical variables. Median differences between time points were computed using the Hodges–Lehmann estimator with their 95% confidence interval. Data were expressed as medians (1st–3rd quartile) and proportions, as appropriate. The non-parametric Friedman test with Dunnett post-hoc correction or Wilcoxon signed ranks test to compare two or more related samples were used, as appropriate. Spearman correlation for non-parametric data was performed.

Statistical analyses were performed using SPSS Software^®^ (Version 23.0). Statistical significance was assumed for two-tailed *p* < 0.05.

## 3. Results

Sixty-six patients with confirmed SARS-CoV-2 infection who underwent RPT had available gas exchange, and only 20 of them presented both data of gas exchange and LUS at the three selected timepoints during RPT and were finally included in this study. Demographic characteristics of the included patients are reported in Table 1.

**Figure 3:**
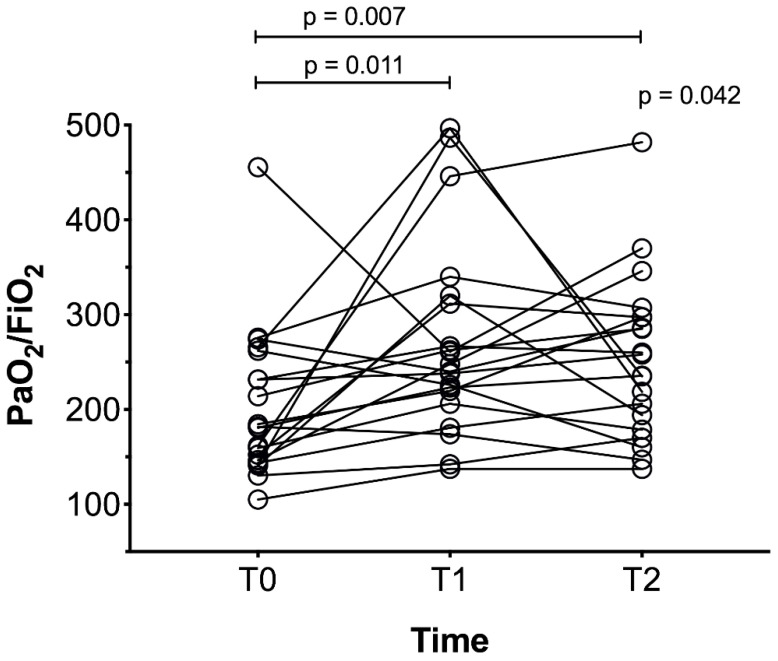
PaO_2_/FiO_2_ at the three timepoints. In this figure all the twenty patients are represented as circles. The response of PaO_2_/FiO_2_ to RPT is represented by a black line during the study time (baseline (T0), immediately after RPT (T1), and 6 h after chest RPT (T2)). Median PaO_2_/FiO_2_ was 181 (105-456), 244 (137-497) and 247 (137-482) at T0, T1, and T2, respectively.

The PaO_2_/FiO_2_ ratio improved at T1 compared to T0 (median difference MD 64 mmHg, 95% confidence interval CI from 9 to 107 mmHg, *p* = 0.011) and at T2 compared to T0 (MD 53 mmHg, 95% CI from 8 to 103 mmHg, *p* = 0.007), as illustrated in Figure 3. To assess the generalizability of our findings, PaO_2_/FiO_2_ ratio response to RPT in a larger ICU-COVID-19 population of 66 patients with available gas exchange data is reported in Figure S2.

**Figure 4:**
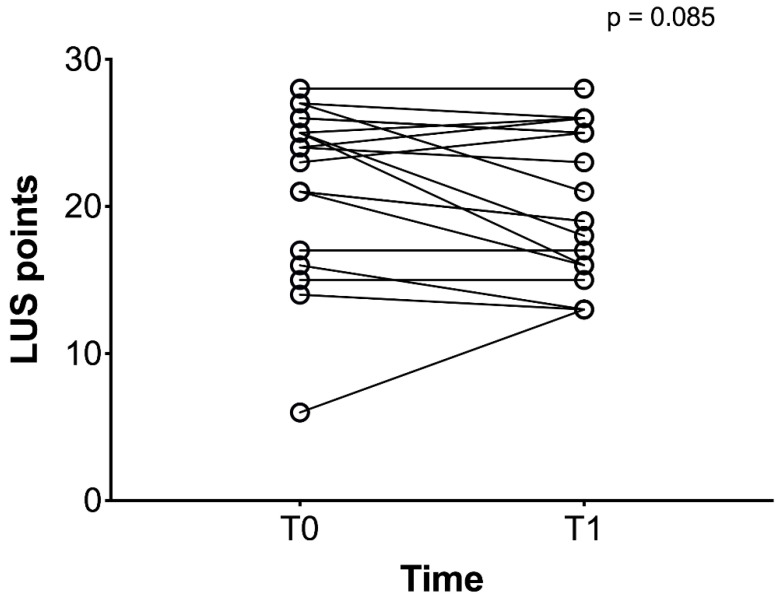
LUS score at baseline (T0) and after RPT (T1). In this figure all the twenty patients are represented as circles. The response of LUS to RPT is represented by a black line during the study time (from T0 to T1).

Overall median LUS score was 24 (6-28) at baseline, and 20 (13-28) after RPT (MD -1, 95% CI from −6 to 2, *p* = 0.085), Figure 4.

Median LUS score of the right lung was 12 (2-14) and 11 (6-14) at baseline and after RPT (*p* = 0.169); while that of the left lung was 12 (3-14) and 11 (3-14) before and after RPT (*p* = 0.260). We could analyze CT scans for a sub-group of N = 14 patients, whose quantitative analysis parameters and correlations with LUS score changes are reported in Table 2.

**Figure 5:**
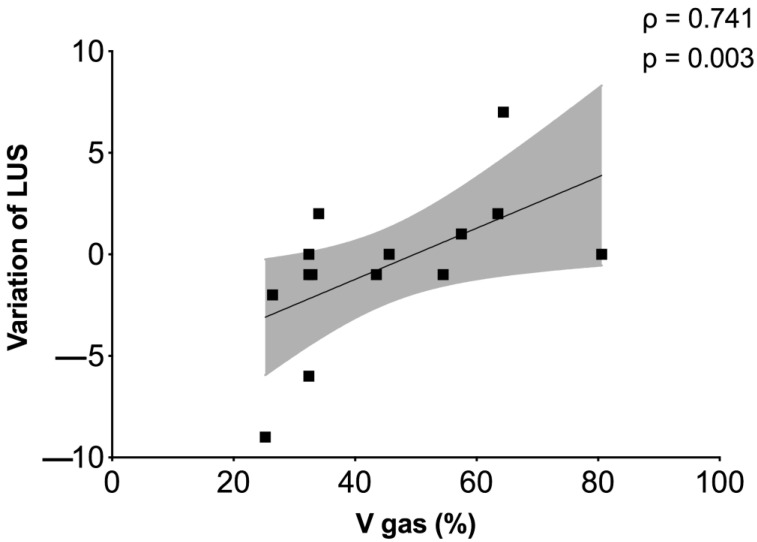
Correlation between LUS and CT scan. Correlation between the volume of gas and hyper-aeration at CT scan and the difference between the first (baseline) and the second (immediately after RPT) LUS have been identified.

We observed a correlation between the variation of LUS score and the % of lung gas volume (ρ = 0.741, *p* = 0.003), as reported in Figure 5.

Hemodynamics parameters before and after chest RPT are reported in Figure S3. Changes in partial pressure of carbon dioxide (PaCO_2_) before and after RPT are reported in the Figure S4.

## 4. Discussion

The main findings of this study are: (1) RPT improves oxygenation in critically ill COVID-19 patients; (2) oxygenation improvement is not reflected by LUS score reduction; (3) the improvement in LUS score after RPT is correlated with the lung gas volume at CT scan; (4) chest RPT does not significantly affect hemodynamics in COVID-19 patients.

Respiratory physiotherapy maneuvers are considered essential for patients’ rehabilitation during ICU stay [10,14], especially as they are effective in improving oxygenation in critically ill patients [6,7] and non-critically ill COVID-19 patients [25]. So far, this was still not investigated in severe COVID-19 patients admitted to ICU who show a different respiratory pattern and distinct response to lung recruitment maneuvers and PEEP [26,27].

To our knowledge, there are few studies investigating the efficacy of RPT and rehabilitation in non-severe COVID-19. In a small randomized controlled trial in elderly COVID-19 patients [28], pulmonary function was significantly improved after six weeks of rehabilitation. However, the early response to RPT maneuvers was not assessed and the ICU COVID-19 patients were not the targeted population.

We therefore proposed an experimental “pre–post” study on COVID-19 patients admitted to the ICU, investigating the early efficacy of RPT on oxygenation and its correlation with quantitative lung imaging parameters. In our study, the ratio between PaO_2_ and FiO_2_ after RPT maneuvers improved substantially and remained higher 6 h thereafter in respect to baseline. Another quasi-experimental study with a “pre–post” design [25] investigated the efficacy of RPT on peripheral oxygenation after a longer period of 4–5 days in non-ICU COVID-19 patients, confirming that RPT maneuvers are effective to improve gas exchange and to reduce oxygen requirement.

In our work, we observed a trend towards higher LUS score improvement in patients with higher oxygenation response, but we did not reach statistical significance. This might be due to the limited sample size, but the peculiar loss of aeration pattern in COVID-19 patients might have affected these findings. While LUS has been evaluated as a diagnostic and prognostic tool in COVID-19 [18], it has an unclear role in assessing aeration changes induced by PEEP changes or other measures that alter lung aeration, such as RPT. The pattern of loss of aeration in COVID-19 is characterized by extensive ground glass opacities that reach the pleural line but also involve peri-hilar regions, which are not assessed by LUS [4]. Radiological findings at CT scan in COVID-19 include ground glass opacities with high perfusion and shunting, lung consolidation, ventilated and hypo-perfused areas, and possible micro-thrombosis. Air bronchograms may follow major consolidations, while pleural effusion is normally absent [29]. Moreover, bedside LUS trends might not catch changes in aeration in the most dorsal regions, where atelectasis areas due to supine positioning or overlapping ventilator-induced lung injury might coexist with the primary viral lung injury.

Four basic patterns at LUS have been identified in COVID-19 as well as in our patients, including a normal pattern (A-lines and <3 B-lines); a mild disease (≥3 B-lines with some confluents, and thickened pleura); B-lines with broken pleural line; and a typical ARDS pattern with subpleural consolidation. The advantages of LUS include the non-invasivity, quick assessment, bedside application, and there being no ionizing radiations [30]. In our study, LUS correlated positively with % of lung gas volume at CT scan. This might suggest that RPT maneuvers could be more effective in patients with higher lung aeration. In fact, our population included patients in different phases of the disease, including those in an advanced weaning period. This finding suggests that LUS could be a supplement to clinical examination to assess aeration [31] before and after RPT at the bedside.

Finally, during respiratory physiotherapy, the systemic hemodynamic was not significantly altered, implying that RPT could be safely applied also in critically ill COVID-19 patients with possible hemodynamic instability. This was not confirmed by a previous work [25] which observed an improvement of heart rate before and after (4–5 days) RPT. However, the different cohorts (non-ICU vs. ICU) and the timing of “post-RPT” assessments (4–5 days vs. immediately after) may explain such distinct hemodynamic responses.

Confirmations may come from an ongoing study in COVID-19 ICU patients which aims to investigate, in a larger cohort, the real benefit of respiratory physiotherapy in severe COVID-19 patients admitted to ICU [32].

In our study, mechanically ventilated patients underwent two distinct protocols of RPT, one for each phase of ICU stay. In general, patients still under assisted mechanical ventilation underwent 30° bed head elevation, subglottic secretion drainage, cough assist maneuver (at zero PEEP), early mobilization, and right lateral to left lateral positioning; while those breathing spontaneously who received COT underwent assisted or stimulated cough maneuvers, sputum induction, positioning, and manual alveolar recruitment, EzPAP, an active cycle of breathing technique, which are typically recognized as airways cleaning techniques [8]. These RPT maneuvers were protocolized before starting this study for reducing biases, and were selected for their proven efficacy in both assisted mechanically ventilated patients and those breathing spontaneously [33,34]. However, despite the indications provided by some national and international guidelines regarding the assumed increased risk of healthcare exposure to the virus [6,10,35], we decided to include sputum induction and subglottic secretion drainage into our protocol. Indeed, recent findings suggested that surface contamination and healthcare exposure following strict decontamination procedures, using personal protective equipment, and negative pressure settings are very limited [36].

This study has several limitations which must be addressed. First, the small sample size because of the COVID-19 pandemic scenario which did not allow us to perform more analyses due to the lack of healthcare resources. Second, the inter-operator variability to interpret different LUS patterns. Third, this was an observational study comprising retrospectively and prospectively collected data, and as stated above, data were collected within the context of the peak of the COVID-19 pandemic with such limitations. Finally, the lack of a control group to assess the efficacy of RPT in COVID-19 ICU patients compared to non-COVID-19 patients.

## 5. Conclusions

This study demonstrated that chest RPT improves oxygenation in severe COVID-19 ICU patients, and that several established RPT techniques can be safely applied in this subgroup of patients to reduce atelectasis. All RPT interventions should be carefully organized, wearing personnel protective equipment to minimize exposure, and planning LUS to assess at bedside the changes in aeration. Further studies are warranted to confirm the efficacy of RPT techniques in COVID-19.

## Figures and Tables

**Table 1:** Characteristics of the included patients who underwent respiratory physiotherapy. Data are expressed as median (1st–3rd quartile) or number (%) as appropriate. BMI, body mass index; SOFA, sequential organ failure assessment; RPT, respiratory physiotherapy; ICU, intensive care unit; COT, conventional oxygen therapy; PSV, pressure support ventilation.

Characteristics of Patients	Included Patients (*n* = 20)
**Demographics**	
Gender, female, *n* (%)	4 (20)
Age, years, median (1st–3rd quartile)	63 (52–75)
BMI, kg/m^2^, median (1st–3rd quartile)	28 (26–30)
**Previous chronic comorbidities, *n* (%)**	
Chronic respiratory diseases	0 (0)
Chronic cardiovascular diseases	2 (10)
Active cancer	0 (0)
Chronic neurologic disorders	0 (0)
Chronic moderate/severe liver diseases	0 (0)
End-stage kidney injury	0 (0)
Chronic hypertension	7 (35)
Diabetes mellitus (type II)	0 (0)
Active smoker	4 (20)
**ICU characteristics, median** (1st–3rd quartile)	
SOFA score at ICU admission	4 (3–4)
Days between symptoms onset and ICU admission	9 (8–14)
Days of mechanical ventilation during ICU stay	18 (11–79)
**Type of ventilation during RPT**, *n* (%)	
PSV	9 (45)
COT	11 (55)
**ICU discharge characteristics**, *n* (%)	
Dead	1 (5)
Alive	19 (95)

**Table 2:** Correlations between variation of LUS score and PaO_2_/FiO_2_ and CT parameters. LUS, lung ultrasound; arterial partial pressure of oxygen (PaO_2_); fraction of inspired oxygen (FiO_2_); V gas, volume of gas.

CT Parameters	Variation of LUS Score (Points)		Variation of PaO_2_/FiO_2_ (mmHg)	
	Spearman ρ	*p*	Spearman ρ	*p*
Volume (mL)	0.240	0.405	−0.267	0.352
Weight (g)	−0.435	0.121	−0.245	0.394
V gas (%)	0.741	0.003 *	0.248	0.391
Mass hyper aerated (%)	0.511	0.064	−0.332	0.244
Mass normal (%)	0.381	0.178	0.444	0.113
Mass poorly aerated (%)	−0.500	0.070	0.486	0.080
Mass non aerated (%)	−0.148	0.610	−0.464	0.096

## Data Availability

Data are available at the corresponding author.

## Supplementary Materials

The following are available online https://www.mdpi.com/article/10.3390/jcm10102139/s1, Figure S1: Areas investigated by Lung Ultrasound (LUS) before and after RPT, Figure S2: PaO_2_/FiO_2_ response to CPT in the overall population of 66 severe COVID-19 ICU patients, Figure S3: Hemodynamic responses to RPT, Figure S4: Changes of PaCO_2_ before and after RPT.

## Collaborators of the GECOVID group

Iole Brunetti^1^, Nicolò Patroniti^1,2^, Francesco Marramao^1,2^, Lara Delpiano^1,2^, Daniele Roberto Giacobbe^3^, Antonio Vena^3^, Lucia Taramasso^4^, Chiara Dentone^4^, Laura Magnasco^4^, Francesca Farina^4^

^1^Anesthesia and Intensive Care, San Martino Policlinico Hospital, IRCCS for Oncology and Neuroscience, Genoa, Italy
^2^Department of Surgical Sciences and Integrated Diagnostic (DISC), University of Genoa, Genoa, Italy
^3^Infectious Diseases Unit, San Martino Policlinico Hospital, IRCCS for Oncology and Neurosciences, Genoa, Italy
^4^Rehabilitation and Functional Education, San Martino Policlinico Hospital, IRCCS for Oncology and Neurosciences, Genoa, Italy
