# Supplementary material for: An Experimental Pre-Post Study on the Efficacy of Respiratory Physiotherapy in Severe Critically III COVID-19 Patients
Source: J Clin Med. 2021 May 15;10(10):2139. doi: 10.3390/jcm10102139 (PMC8156952; doi:10.3390/jcm10102139)

Supplemental Material (SM)

An Experimental Pre-Post Study on the Efficacy of Respiratory Physiotherapy in Severe Critically Ill COVID-19 patients

Denise Battaglini[1,2], Salvatore Caiffa[3], Giovanni Gasti[1,4], Elena Ciaravolo[1,4],

Chiara Robba[1], Jacob Herrmann[5], Sarah E. Gerard[6], Matteo Bassetti[7], Paolo Pelosi[1,4] and

Lorenzo Ball[1,4]

for the **GECOVID**.

[1] Anesthesia and Intensive Care, San Martino Policlinico Hospital, IRCCS for Oncology and Neuroscience, 16132, Genoa, Italy

[2] Department of Medicine, University of Barcelona (UB), 08007, Barcelona, Spain

[3] Intensive Care Respiratory Physiotherapy, Rehabilitation and Functional Education, San Martino Policlinico Hospital, IRCCS for Oncology and Neurosciences, 16132, Genoa, Italy

[4] Department of Surgical Sciences and Integrated Diagnostics (DISC), University of Genoa, 16132, Genoa, Italy

[5] Department of Biomedical Engineering, Boston University, Boston, MA 02215, USA

[6] Department of Radiology, University of Iowa, Iowa City, IA 52242, USA

[7] Infectious Diseases Unit, San Martino Policlinico Hospital, IRCCS for Oncology and Neurosciences, 16132, Genoa, Italy

SM Contents

Lung ultrasound score, [page 2]

Figure S1. Areas investigated by Lung Ultrasound (LUS) before and after CPT, [page 3]

Figure S2. PaO_2/FiO_2 response to CPT in the overall population of 66 severe COVID-19 ICU patients, [page 4]

Figure S3. Hemodynamic responses to CPT, [page 4]

Figure S4. Changes of $PaCO_2$ before and after CPT, [page 4]

Lung ultrasound score

Six specific regions for each lung were identified and categorized by the following aeration patterns:

A = Lung ultrasound of a normal aerated lung is defined as the presence of A lines at the same distance from skin to pleural line; B = multiple B lines as hyperechoic artifacts, with a "comet tail" that arises from the pleural line to the lung parenchyma. B lines are classified in 1) B1 lines (associated with an interstitial syndrome with reduced lung aeration); 2) B2 lines (confluent lines that can converge appearing as "with lung" or "glass-rockets", that is the equivalent of ground-glass at CT-scan [1]); C = Lung consolidation is characterized by hepatization of the lung parenchyma, with or without air bronchogram. Points for each pattern: A= 0 point, B1= 1 point, B2= 2 points, C= 3 points. The maximum LUS score to be achieved was 36, while the minimum 0. The higher score, the lower aeration [2].

References

1. Man, M.A.; Dantes, E.; Domokos Hancu, B.; Bondor, C.I.; Ruscovan, A.; Parau, A.; Motoc, N.S.; Marc, M. Correlation between Transthoracic Lung Ultrasound Score and HRCT Features in Patients with Interstitial Lung Diseases. *J Clin Med* **2019**, *8*, 1199, doi:10.3390/jcm8081199.
2. Bouhemad, B.; Mongodi, S.; Via, G.; Rouquette, I. Ultrasound for "Lung Monitoring" of Ventilated Patients. *Anesthesiology* **2015**, *122*, 437–447, doi:10.1097/ALN.0000000000000558.

Figure S1. Areas investigated by Lung Ultrasound (LUS) before and after CPT.

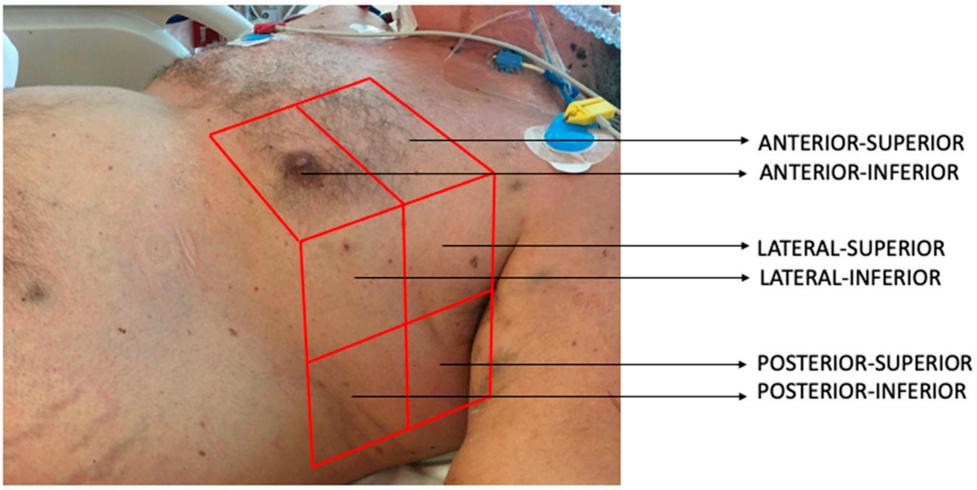

Figure S2. PaO_2/FiO_2 response to CPT in the overall population of 66 severe COVID-19 ICU patients.

The PaO_2/FiO_2 ratio improved at T1 compared to T0 (median difference MD 52 mmHg, 95% confidence interval CI from 22 to 62 mmHg, $p < 0.0001$) and at T2 compared to T0 (MD 31 mmHg, 95% CI from 3 to 59 mmHg, $p < 0.0001$).

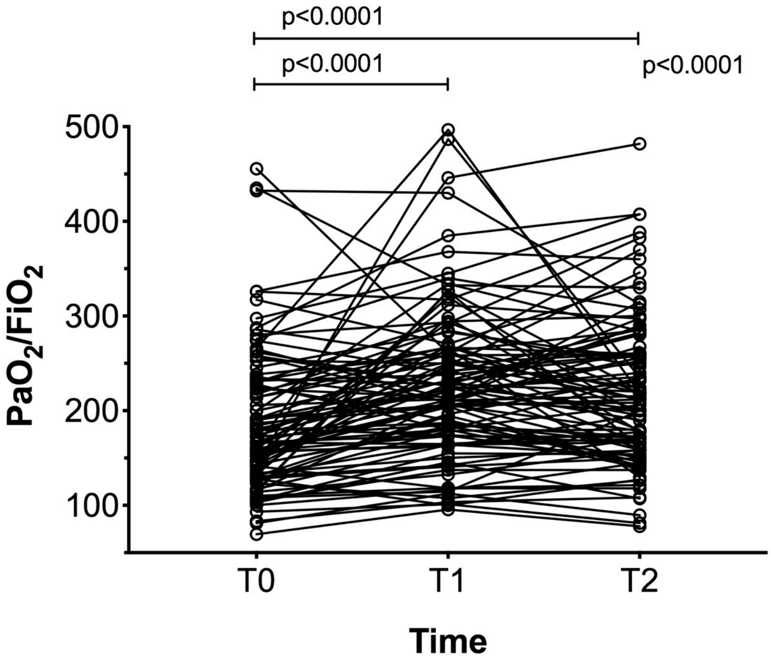

Figure S3. Hemodynamic responses to RPT.

Hemodynamic changes in heart rate (HR) and mean arterial pressure (MAP) are reported. Median (IQR) HR was 86 (80-130), 90 (64-120), and 88 (66-114) at T0, T1, and T2, respectively ($p = 0.307$). Median (IQR) MAP was 88 (70-121), 92 (67-117), and 90 (73-100) at baseline, after CPT and 6 hours thereafter, respectively ($p = 0.127$).

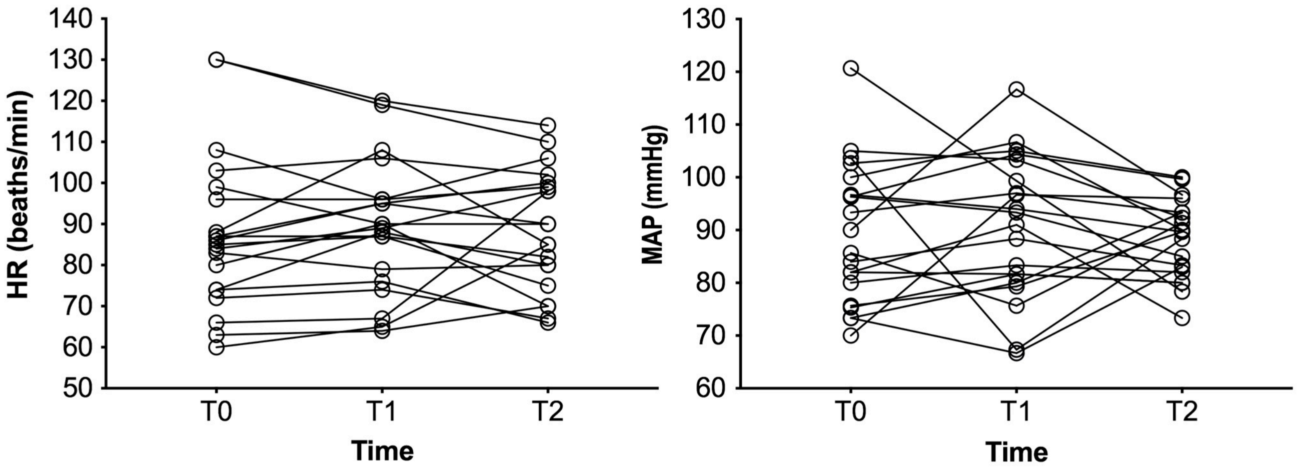

Figure S4. Changes in partial pressure of carbon dioxide ($PaCO_2$) before and after CPT.

Median (IQR) $PaCO_2$ was 41.5 (31-57), 41.5 (29-64), and 40.5 (30-61) at baseline, after CPT and 6 hours thereafter, respectively ($p = 0.669$).

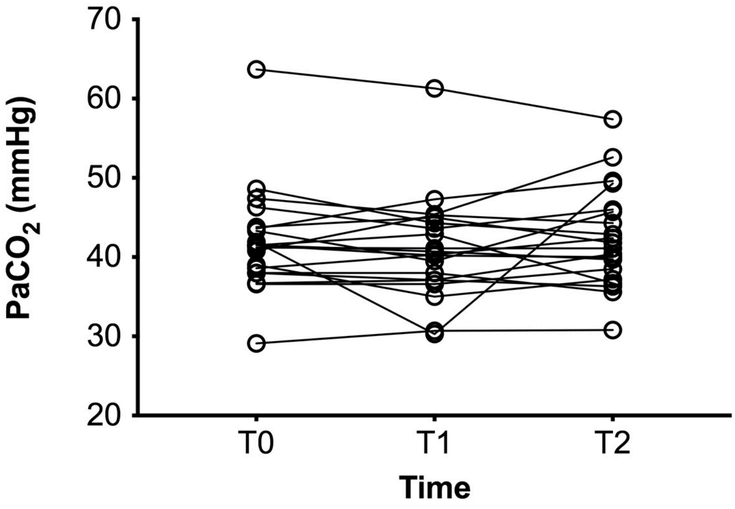

Supplement: Supplementary file 1 [file jcm-10-02139-s001.zip › jcm-1217330-supplementary.pdf]
